# CYP3A5 and ABCB1 genotype influence tacrolimus and sirolimus pharmacokinetics in renal transplant recipients

**DOI:** 10.1186/s40064-015-1425-5

**Published:** 2015-10-23

**Authors:** Yi Li, Lin Yan, Yunying Shi, Yangjuan Bai, Jiangtao Tang, Lanlan Wang

**Affiliations:** Department of Clinical Immunological Laboratory, West China Hospital, Sichuan University, Chengdu, 610041 The People’s Republic of China; Department of Nephrology, West China Hospital, Sichuan University, Chengdu, 610041 The People’s Republic of China

**Keywords:** Renal transplantation, Polymorphism, CYP3A5 and ABCB1, Tacrolimus, Sirolimus, Pharmacokinetics

## Abstract

CYP3A5 and ABCB1 polymorphisms have been shown to influence tacrolimus blood concentrations and dose requirements, but the conclusion in the current reports were inconformity. Sirolimus are also metabolized by CYP3A subfamily and are substrates of the P-gp. The aim was to determine whether these polymorphisms affect tacrolimus (TAC) and sirolimus (SRL) trough concentrations and dose requirements after renal transplantation. 153 renal transplant recipients were enrolled into this study, 112 were treated with TAC-based regimen, Another 43 recipients received SRL-based regimen. The recipients’ mean follow-up time was 20 mo (range 15–27 mo). All renal transplant recipients were all in a stable stage. The trough concentration and daily dose of TAC and SRL were gained from each recipient. All recipients were genotyped for CYP3A5 (6986A>G), CYP3A4 intron 6 (CYP3A4*22), CYP3A4*18, ABCB1 exon 26 (3435C>T), exon 12 (1236C>T) and 2677G>T/A SNPs by HRM analysis (high-resolution melting curve analysis). The TAC and SRL concentration/dose ratio (C/D) in recipients with CYP3A5 (*)3/(*)3 were significantly higher than that of those with (*)1 allele (P < 0.05). However, there was no significant correlation between adjusted TAC and SRL trough concentrations or dose requirements with CYP3A4 and ABCB1 SNPs genetic polymorphisms. In recipients with TAC-based or SRL-based therapy, the CYP3A5 genes (6986A>G) can influence the TAC and SRL pharmacokinetics in renal transplant recipients.

## Background

The calcineurin inhibitors (CNI), tacrolimus (TAC) are the most widely used immunosuppression drugs to prevent allograft rejection after solid organ transplantation. Both these drugs display a narrow therapeutic index and high inter-individual pharmacokinetic variability, so monitoring their blood levels is required to avoid rejection and reduce toxicity (López-Montenegro Soria et al. 2010). Sirolimus (SRL) is another immunosuppressant by replacing the CNIs to prevent progression in chronic kidney disease (CKD) following organ transplantation. SRL pharmacokinetics also exhibits wide subject variability (Sam et al. 2011).

The calcineurin-inhibitor TAC and SRL undergo extensive first-pass metabolism in the human liver. This process is catalyzed by cytochrome P450 (CYP) 3A enzymes. CYP3A4 and CYP3A5 have been identified as the major enzymes responsible for the metabolism of CNI and SRL in CYP3A subfamilies. On the other side, both CNI and SRL are the substrates of P-gp, an efflux transporter encoded by the MDR1/ABCB1 [adenosine triphosphate (ATP) binding cassette subfamily B, member 1] gene, which actively transports common drugs from the intracellular to the extracellular domain and thereby influencing their pharmacokinetics (Sakaeda et al. 2003).

Studies reported that the single-nucleotide polymorphisms (SNPs) of genes encoding CYP3A4, CYP3A5 and ABCB1 could be important determinants of CNI and SRL bioavailability and metabolism (Sakaeda et al. 2003; Lukas et al. 2010; Mourad et al. 2006; Li et al. 2006). In the general population, it is estimated that genetics accounts for 20–95 % of the variability in drug disposition and effects (Evans and McLeod 2003). Some of the most commonly studied ABCB1, CYP3A4 and CYP3A5 variants include ABCB1 1236C>T, 2677G>T/A, 3435C>T, CYP3A4 392A>G and CYP3A5 6986A>G. Among all those variants, the polymorphic CYP3A5 6986A>G plays a major role. The G>A mutation in intron three results in a splice defect of the mRNA and produces an unstable and non-functional protein. The mutated allele was named CYP3A5*3 and the wild type was assigned CYP3A5*1. Those recipients bearing at least one active CYP3A5*1 required higher doses of tacrolimus to achieve therapeutic plasma concentrations than CYP3A5 non-expressors (MacPhee et al. 2005). The ABCB1 1236C>T and 3435C>T are synonymous SNPs, those two SNPs were correlated with ABCB1 function and resulted in substrate specificity changes (Letourneau et al. 2005; Brinkmann 2002). The impact of ABCB1 polymorphisms to the inter-individual variability in the blood concentration of CNI and SRL were partly controversially discussed.

The acute rejection was gonging down by the introduction of immunosuppression drugs, while the longtime survival rate was not increasing. The reason was the drug overuse and drug toxicity after longtime using. Therefore, we performed a clinical study to investigate the impact of the CYP3A5, CYP3A4 and ABCB1 genotypes on the dose requirement and pharmacokinetics of CNI and SRL in stable kidney transplant recipients receiving maintenance treatment, aimed to draw up individual treatment regimen for every recipient and reduced drug toxicity.

## Methods

### The inclusion of renal transplant recipients

This study was approved by the Ethics Committee of Chinese Human Genome and the Ethics Committee of West China Hospital, and written informed consent was obtained from all recipients. We included 112 recipients who received a first kidney graft in West China Hospital. The TAC and SRL treated recipients were received the induction therapy with alemtuzumab. Among all the renal transplant recipients, 112 recipients received a tacrolimus (TAC, Prograf) based regimen (TAC+MMF+steroid), The initial oral daily dose of tacrolimus was 0.04 ~ 0.08 mg/(kg*d). The daily dose was adjusted according to the blood trough tacrolimus concentration (C0); the target trough concentration was 5–10 ng/mL. After 3 months, target C0 was decreased to 3–6 ng/mL for long-term maintenance. In addition to tacrolimus, the recipients received MMF and steroids at a standard dosage regimen. 1500 mg/d MMF was given as two equally divided doses. The steroid regimen was 1000 mg of i.v. methylprednisolone at the time of surgery and 500 mg/d i.v. the next 2 d. This schedule was followed by 80 mg/d of oral prednisone, progressively tapered by 10 mg/d to 20 mg per day until 3 months after transplantation, reduced to a maintenance dose of 5 mg daily or withdrawn. All recipients received tacrolimus based regimen for more than 1 month. All the recipients have ever happened acute rejection.

The other 43 recipients received a sirolimus (SRL, Rapamune) based regimen (SRL+MMF+steroid), The initial oral daily dose of sirolimus was 0.04 ~ 0.06 mg/(kg*d). Steroid and MMF therapy was the same as the tacrolimus regimen. SRL dose was adjusted to maintain a blood trough level of 5–10 ng/mL.

The recipients received sirolimus based regimen or TAC based regimen for more than 1 month and in stable state without rejection. The demographic and clinical characteristics of the recipients were listed in Table 1.

**Table 1:** Demographic and clinical characteristics of renal transplant recipients treated by TAC and SRL

	TAC	SRL
Number (N)	112	43
Age (years)	35 (31–46)	35 (34–46)
Sex (male/female)	70/42	30/13
Therapy time (month)	11 (7–20)*	6 (4–10)
Concentration (ng/mL)	4.5 (3.9–5.8)	5.9 (3.5–8.3)
Glucose (mmol/L)	4.81 (4.6–5.2)	5.09 (4.86–5.77)
Systolic pressure (mmHg)	132 (126–140)	126 (110–138)
Diastolic pressure (mmHg)	98 (67–117)	87 (65–106)
ALT (IU/L)	18.6 (14.2–25.8)*	26 (18–37.5)
AST (IU/L)	23 (16.3–26)*	25.5 (20.5–36.2)
Cholesterol (mg/dL)	4.67 (3.97–5.51)*	5.4 (4.92–5.77)
Triglyceride (mg/dL)	1.23 (0.98–2.14)	1.68 (1.09–2.21)
Creatinine (mg/dL)	1.4 (1.08–2.14)*	1.20 (1.06–1.76)
Proteinuria (g/mL)	0.2 (0.1–0.5)	0.3 (0.2–0.7)
Imunosuppressive	TAC/MMF/	SRL/MMF/
Regimen	Prednisolone	Prednisolone

The datas were expressed as median (range)

* P < 0.05 compared with SRL group

### Genomic DNA extraction

Blood samples (3 mL) were collected in EDTA tubes and genomic DNA was isolated from whole blood samples using the whole blood DNA kit (Biotake corporation, Beijing, China). DNA was extracted from 200 µL of the whole bloods according to the manufacturer’s protocol. The concentration of DNA was diluted to 10 ng/µL for working solutions and the isolated DNA was stored at −20 °C.

### Polymerase chain reaction and high-resolution melting method

CYP3A5, CYP3A4 and ABCB1 SNPs were assessed. Some samples were previously genotyped by sequencing as controls for the SNPs. The polymerase chain reaction (PCR) and melting curve analyses were performed under the same conditions in a 96-well plate on the Light Cycler480 (Roche Diagnostics, Penzberg, Bavaria, Germany). The primers were designed into a small fragment surrounding the polymorphisms and avoiding the presence of other sequence variations in the primer region. The reaction mixtures contained 1.0 µL purified genomic DNA (10 ng/µL), 0.1 µL forward primer, 0.1µL reverse primer, 5µL Roche Mix (including 20 × EVA-GREEN, dNTP, Hot Star Taq^®^ Plus DNA Polymerase, 10 × buffer), 1.4 µL 25 mM Mgcl_2_ and 2.4 µL H_2_O. Real-time PCR was performed under the following conditions: an initial denaturation step at 95 °C for 15 min, continued with 50 cycles of 95 °C for 10 s, 60 °C for 15 s, and 72 °C for 20 s. After the amplification phase, a melting curve analysis was performed at 95 °C for 1 min, 40 °C for 1 min, 65 °C for 1 s, followed by slow heating at 0.01 °C/s to 95 °C. Collected data were analyzed by the LightCycler 480 Gene Scanning software v1.2 (Roche Diagnostics). Temperature shifting by selecting threshold, then automatic grouping by calculation. The exactly same setting of the normalization was used for all experiments. The genotype of subset was defined according to known genotypes of controls.

### Immunosuppression drug blood concentration and other laboratory tests measurement

CNI (tacrolimus and cyclospine) dosage was given twice daily in equal amounts at 08:00 and 20:00 h. Blood samples were collected exactly before morning doses of CNI for determination of trough blood concentrations (C0). Sirolimus dosage was given once daily and the trough concentration was tested before taking drug. Those drug concentrations were tested by an automated enzyme immunoassay (SIEMENS V-Twin) analyzer. Dose-adjusted trough blood concentrations (C/D ratio) were calculated by dividing the C0 (ng/mL) by the corresponding 24-h dose per kilogram basis (mg/kg body weight). We obtained serum levels of Cystatin-C, alanine aminotransferase (ALT), alkaline phosphatase (ALP), bilirubin, and creatinine (Roche Diagnostics, Roche, Switzerland), along with the trough level of those drugs.

### Statistical analysis

Data were expressed as mean ± SD or median (range). Analysis was performed using SPSS 16.0 statistical software (SPSS Inc., Chicago, IL, USA). The Chi-square test was used to examine the difference in categorical data. The Mann–Whitney U test and Kruskal–Wallis test were employed to determine the difference in continuous values among groups. The allele and genotype frequencies of the CYP3A and ABCB1 polymorphisms were assessed for deviation from the Hardy–Weinberg equilibrium using the Chi-square test. A P value of <0.05 was considered statistically significant.

## Results

### The demographic and clinical characteristics of the recipients

There was no significant difference in age and gender among the three groups (Table 1). The recipients used CNI for a longer time than the recipients used sirolimus. After using those drug for over 1 month, the drug concentration in those recipients were stable, with 4.5 (3.9–5.8) ng/mL for TAC and 5.9 (3.5–8.3) ng/mL for SRL.

Most of the renal transplant recipients used SRL were initially considered candidates for TAC to SRL conversion because of renal dysfunction. After conversion to SRL for more than 1 month, the renal function has turned better for those recipients, the SCr was lowest in SRL group 1.20 (1.06–1.76) mg/dL among TAC group 1.43 (1.08–2.14) mg/dL, without a significant increasing in proteinuria (P < 0.05). In the mean time, the blood pressures (systolic pressure and diastolic pressure) were higher in CNI regimen treated group (P > 0.05).

On the other side, sirolimus has its own side effects. Firstly, we found that the recipients used sirolimus occurred hyperlipemia with significantly increasing in Cholesterol than the recipients used CNI. Second, ALT and AST level in those recipients used SRL were significantly higher than the recipients used CNI, although in the normal range (P < 0.005). (Table 1).

### Tacrolimus

Among the 112 TAC treated recipients,there were 52 CYP3A5 6986A>G*1/*1 and *1/*3 (expressors) genotype and 60 *3/*3 (non-expressors) genotype. The CYP3A5*1/*1 and *1/*3 genotypes were combined, as these represent the CYP3A5 expressers category, we found no significant difference between the CYP3A5*1/*1 and *1/*3 genotypes groups and *3/*3 genotype was kept in the non-expressers category. The C/D ratios in *1/*1+*1/*3 recipients [124(113–165.4)] were much lower than *3/*3 recipients [184 (148–261)] (P < 0.05, Table 2). In the mean time, the recipients with CYP3A5*1 allele (expressor) need higher TAC dose [2.7 (1.7–2.9)] than *3/*3 recipients [2.3 (1.6–2.9)], (P < 0.05, Table 2). When multiple binary logistic regression analysis was carried out to assess the baseline clinical covariates (age, gender, BMI of recipients, HLA, preoperative creatinine and urine volume, dialysis time), it did not modify the association between the CYP3A5 genotype and the association of C/D ratios (P = 0. 03),

**Table 2:** The effects of CYP3A4, CYP3A5 and ABCB1 genetic polymorphisms on tacrolimus and sirolimus daily requirements, concentration/dose ratio after renal transplantation

	Tacrolimus				Sirolimus			
	N	Daily dose (mg/kg/day)	Concentration (ng/mL)	C/D ratio (ng/mL)/(mg/kg/day)	N	Daily dose (mg/kg/day)	Concentration (ng/mL)	C/D ratio (ng/mL)/(mg/kg/day)
CYP3A5 (6986A>G)	112				43			
*1/*1+*1/*3	52	2.7 (1.7–2.9)	6.2 (5.6–7.4)	124 (113–165.4)	20	1.28 (1.00–1.56)	4.2 (5.75–7.2)	249 (248.7–409.6)
*3/*3	60	2.3 (1.6–2.9)*	6.8 (4.5–8.7)	184.4 (148–261.7)*	23	1.00 (1.00–1.00)*	4.0 (5.7–7.7)	389.1 (293.7–537.9)*
ABCB1 3435C>T								
CC	30	1.0 (1.0–3.2)	4.3 (1.9–6.8)	242.1 (110.2–374)	18	1.00 (1.00–1.00)	7.1 (5.1–7.6)	364.5 (310.6–574.3)
CT	62	1.25 (1.3–2.1)	6.2 (4.1–7.5)	165.2 (115.9–205)	20	1.10 (1.00–1.20)	6.2 (3.3–7.8)	365.6 (177.6–498.6)
TT	20	1.5 (1.0–2.0)	7.1 (5.5–10.2)	324 (214.1–559)	5	1.00 (0.65–1.5)	4.3 (3.75–6.75)	316.5 (217.9–549.8)
ABCB1 1236C>T								
CC	17	1.0 (1.0–2.0)	6.0 (3.7–9.5)	247 (104.6–441)	8	1.00 (1.00–1.75)	5.7 (3.0–7.1)	298.6 (128.6–364.6)
CT	50	1.5 (1.0–2.0)	7.1 (5.7–8.5)	181.1 (142.8–268)	20	1.00 (1.00–1.50)	7.5 (4.8–7.9)	385.9 (264.8–628.9)
TT	45	1.3 (1.1–2.0)	5.0 (3.9–5.4)	214 (167–245)	15	1.00 (0.9–1.00)	5.2 (4.1–7.5)	381.9 (278.6–48.9)
ABCB1 2677C>T/A								
CC	22	1.0 (1.0–2.0)	6.0 (3.7–9.5)	217 (104.6–441)	7	1.00 (1.00–1.75)	5.7 (3.0–7.1)	298.6 (128.6–364.6)
CT	49	1.15 (1.0–2.0)	7.1 (5.7–8.5)	161.1 (142.8–268)	16	1.00 (1.00–1.50)	6.5 (4.8–7.9)	386.9 (264.8–628.9)
TT	16	1.25 (1.1–2.0)	5.0 (3.9–5.4)	204 (167–245)	11	1.00 (0.9–1.00)	4.2 (4.1–7.5)	371.9 (278.6–48.9)
A	25	1.3 (1.1–2.0)	5.0 (3.9–5.4)	214 (167–245)	9	1.00 (0.9–1.00)	5.2 (4.1–7.5)	341.9 (278.6–48.9)
CYP3A4 (CYP3A4*22)								
CC	112	2.0 (1.0–2.0)	6.5 (5.2–8.7)	205.2 (126.9–324)	43	1.07 (0.86–1.65)	6.1 (5.3–7.8)	364.8 (248.7–525.8)
CYP3A4 (CYP3A4*18)								
TT	110	2.0 (1.0–2.0)	6.5 (5.2–8.7)	205.2 (126.9–324)	43	1.07 (0.86–1.65)	6.1 (5.3–7.8)	364.8 (248.7–525.8)
CT+TT	2	2.0/3.0	7.0/6.4	214/323	0	/	/	/

The datas were expressed as median (range)

* P < 0.05 compared with *1/*1+*1/*3

For the ABCB1 3435C>T, there were 30 CC genotype,62 CT genotype and 20 TT genotype. There was no significant difference between different genotype. For the ABCB1 1236 C>T, with 17 CC genotype, 50 CT genotype, 45 TT genotype. For the ABCB1 2677 C>T/A, with 22 CC genotype, 49CT genotype, 16 TT genotype and 25 with A genotype. We did not found any association between ABCB1 SNPs and TAC pharmacokinetics index.

For the CYP3A4 intron 6(CYP3A4*22), There was only one genotype CC in Chinese renal transplant recipients.

For the CYP3A4*18, there was only 2 mutation was found and there number was too small to calculate.

### Sirolimus

Among the 43 SRL treated recipients,there were 20 CYP3A5 6986A>G*1/*1 and *1/*3 (expressors) and 23 *3/*3 (non-expressors). The dose/trough concentration ratios in *1/*1 and *1/*3 recipients [249 (248–409.6)] were much lower than *3/*3 recipients [389.1 (293.7–537.9)] (P < 0.05). In the mean time, the recipients with CYP3A5*1 allele (expressor) need higher TAC dose [1.28 (1.00–1.56)] than *3/*3 recipients [1.0 (1.0–1.0)], (P < 0.05, Table 2). When multiple binary logistic regression analysis was carried out to assess the baseline clinical covariates (age, gender, BMI of donors and recipients, HLA, PRA, preoperative creatinine and urine volume, dialysis time), it did not modify the association between the CYP3A5 genotype and the association of C/D ratios (P = 0. 02).

For the ABCB1 3435C>T, there were 18 CC genotype, 20 CT genotype and 5 TT genotype. There was no significant difference between different genotype. For the ABCB1 1236 C>T, with 8 CC genotype, 20 CT genotype,15 TT genotype. For the ABCB1 2677 C>T/A, with 7 CC genotype, 16 CT genotype, 11 TT genotype and 9 with A genotype. We did not found any association between ABCB1 SNPs and TAC pharmacokinetics index.

For the CYP3A4 intron 6(CYP3A4*22) and CYP3A4*18, There was only one genotype CC in Chinese renal transplant recipients.

### Renal function

We found no significant effect of the recipients CYP3A5 and ABCB1 genotypes on the eGFR of TAC or SRL treated regimen.

## Discussion

Tacrolimus and sirolimus have become important immunosuppressive drugs in the field of organ transplantation. However, both those drugs exhibit a low bioavailability and there is a large inter-individual variability in their pharmacokinetics. The pharmacogenetics of genetic polymorphisms in drug metabolizing gene played an important role in interindividual differences of drug bioavailability.

### Tacrolimus

In this study, we found CYP3A5 played an important role in the pharmacokinetics of TAC. The dose of tacrolimus was higher in the recipients with CYP3A5*1 allele (expressor) than the recipients with CYP3A5*3 allele (non-expressor), and the concentration/dose ratio was lower in CYP3A5*1 allele (expressor). Several studies have observed the association between this CYP3A5 gene polymorphism and the tacrolimus dose requirement and indicate that CYP3A5 expressers require a larger tacrolimus dose in order to maintain the same blood concentration (Rong et al. 2010; Singh et al. 2009; Renders et al. 2007; Kuypers et al. 2007; Zhang et al. 2005). It is important to draw up different treatment plan for different recipients of different race.

The starting dose for the recipients was subsequently adjusted according to blood concentration in clinical, but the problem was that CYP3A5 expressers exhibited a mean tacrolimus blood level much lower than the target. Conversely, the non-expressers had higher tacrolimus trough concentrations than the target concentration, which may induce side effects such as hyperglycemia, nephrotoxicity and infection. In our study, we found the expressers need 1.3 ~ 1.5 fold dose than the unexpressers in Chinese renal transplant recipients (Zhang et al. 2005). CYP3A5 genotype analysis could be performed prior to transplantation in order to help the initial dosing of tacrolimus.

The results from previous studies on the relationship between the ABCB1 polymorphism and tacrolimus pharmacokinetics are controversial. The present study we have not found a significant influence of ABCB1 C3435T polymorphism on tacrolimus dose requirement for renal transplant recipients. Several previous studies have drawn similar conclusions that the genotype or haplotype of ABCB1 influences the tacrolimus dose requirement. Subsequent studies, however, fail to prove such an association (Mourad et al. 2006; Li et al. 2006; Rong et al. 2010; Haufroid et al. 2006).

### Sirolimus

The study on sirolimus was limited by the smaller sample size. Similar to tacrolimus, the dose/trough ratio was lower in CYP3A5-expressors compared with non-expressors.The mean higher dosage administered in this group. The data on the impact of CYP3A5 on sirolimus dose requirement, however, are contradictory (Lukas et al. 2010; Renders et al. 2007; Mourad et al. 2005; Le Meur et al. 2006; Miao et al. 2008; Zochowska et al. 2012). Recently, Le Meuer et al. (Le Meur et al. 2006) confirmed that CYP3A5 expressors require significantly higher sirolimus doses to reach the C0 target range than non-expressors. Accordingly, Anglicheau et al. demonstrated that sirolimus concentration/dose ratio was lower in CYP3A5 expressors than in non-expressors, whereas Mourad et al. (Mourad et al. 2005) showed no significant correlation between adjusted sirolimus trough concentrations or dose requirements and genetic polymorph-isms. Genetic variants of efflux transporters seemed to play a minor role, as there was no significant influence of ABCB1 or ABCC2 polymorphisms on the pharmacokinetics of sirolimus (Hebert et al. 2003; Hesselink et al. 2004; Elens et al. 2014; Hesselink et al. 2014).

In conclusion, CYP3A5*3 SNP was significantly correlated with TAC and SRL dose requirement and dose-adjusted concentrations in renal transplant recipient s of China. CYP3A5 expressers were associated with lower C/D rations and thereby require higher TAC and SRL dose to achieve therapeutic concentration compared with non-expressors. Performing CYP3A5 genotyping prior to transplantation is helpful for drug selection and dosing and thus could contribute to a better-individualized immunosuppressive therapy.
